# Optimal Optic Nerve Sheath Diameter Threshold for the Identification of Elevated Opening Pressure on Lumbar Puncture in a Chinese Population

**DOI:** 10.1371/journal.pone.0117939

**Published:** 2015-02-09

**Authors:** Lijuan Wang, Liangshu Feng, Yan Yao, Yuzhi Wang, Ying Chen, Jiachun Feng, Yingqi Xing

**Affiliations:** 1 The Neuroscience Center, Department of Neurology, The First Hospital of Jilin University, Jilin University, Changchun, China; 2 Department of Epidemiology and Biostatistics, School of Public Health, Jilin University, Changchun, China; Save Sight Institute, AUSTRALIA

## Abstract

Ultrasonography of the optic nerve sheath diameter (ONSD) is a non-invasive and rapid method that might be helpful in the identification of increased intracranial pressure (ICP). The use of an ONSD greater than 5 mm on ultrasound as an indicator of increased ICP in a Caucasian population has been studied. However, the cut-off point of this predictor in Chinese patients has not been established. Thus, we conducted this study to identify the ONSD criterion for the detection of elevated opening pressure on lumbar puncture (LP) in a Chinese population and to investigate the influencing factors. This study was a blind cross-sectional study. Patients who presented with suspected increased ICP were included. The opening pressure on LP of each participant was confirmed. We analyzed the clinical differences between the groups of patients with abnormal and normal opening pressures on LP. A receiver operating characteristic curve was constructed to determine the ONSD cut-off point for the identification of abnormal opening pressure on LP. In total, 279 patients were recruited, and 101 patients presented with elevated opening pressure on LP. ONSD was a significant independent predictor of elevated opening pressure on LP (p<0.001). However, no statistical significance was observed regarding the factors that might have affected this relationship including gender, age, body mass index, waistline, head circumference, hypertension and pathological subtype. The ONSD cut-off point for the identification of elevated opening pressure on LP was 4.1 mm; this cut-off yielded a sensitivity of 95% and a specificity of 92%. ONSD is a strong and accurate predictor of elevated opening pressure on LP. The cut-off point of this predictor in a Chinese population was remarkably lower than that found in a Caucasian population. Thus, ethnic differences should be noted when using the ONSD as an indicator of increased ICP.

## Introduction

Increased ICP is considered to be an acute situation that is associated with poor clinical outcomes, such as high rates of mortality in various neurological diseases [1,2]. Invasive ICP monitoring remains the gold standard for the diagnosis of ICP [3]. However, due to the invasiveness of ICP monitoring, which can result in complications such as hemorrhage and bacterial colonization [4], this technique should not be applied to all patients with increased ICP. Additionally, a lack of neurosurgeons and contraindications, such as coagulopathy or thrombocythemia, might reduce the feasibility of invasive ICP monitoring. Moreover, intracranial devices are invasive. Thus, noninvasive, simple, reproducible, bedside methods for the assessment of increased ICP are urgently needed [5]. Ocular ultrasonography has recently been used to assess increased ICP [6–9]. This technique was developed based on ocular anatomy. The optic nerve, which is as an outward form of the diencephalon during embryogenesis, is wrapped by a nerve sheath that is derived from three layers of meninges and protrudes toward the orbit. As a consequence of this communication, cerebrospinal fluid (CSF) can transfer freely between the intracranial and intraorbital subarachnoid space [10]. The intraorbital subarachnoid space, which surrounds the optic nerve, is subject to the same pressure changes as the intracranial subarachnoid space [11]. A linear relationship between the ICP and the subarachnoid pressure of the optic nerve has previously been confirmed in fresh cadavers [12]. Next, the linear covariance of the CSF pressure with the ONSD was confirmed [13]. Subsequently, the ONSD was observed to be positively related to increased ICP using optic nerve sonography and magnetic resonance imaging (MRI) in several western studies [14,15]. Nevertheless, the diagnostic criteria for increased ICP based ultrasonographic measures of ONSD have not been established. Several studies have reported a cut-off point of 5 mm for the identification of increased ICP or elevated opening pressure on LP [15–17]. Thus far, the majority of the published studies have involved small sample sizes [18] and were performed in Caucasian populations. Additionally, little is known about the factors that potentially affect the relationship between the ONSD and ICP. Moreover, whether the upper limits of normal ONSDs vary across different races remains unknown. It has been reported that LP pressures above 20 cm H_2_O when the patient is relaxed with straight legs reflects the presence of increased ICP [19]. Thus, we performed this study to identify the ONSD criterion for the detection of elevated opening pressure on LP in a Chinese population and to investigate the factors that potentially influence this relationship.

## Materials and Methods

### Study Setting

The study was performed in the First Hospital of Jilin University, which is a general public hospital in China. The Ultrasound Center in the Department of Neurology is one of four ultrasound training centers in China.

### Patients

This was a blind cross-sectional study that recruited patients who were suspected of having increased ICP for various reasons and underwent lumbar puncture between March 2013 and December 2013. The exclusion criteria were the following: 1) aged<18 years old, 2) a history of glaucoma or current medications that might have affected CSF pressure, and 3) ophthalmic diseases, such as tumors or traumas. Each participant underwent a lumbar puncture to confirm the ICP status. The following patient data were recorded: age, sex, body mass index (BMI), waistline, head circumference, systolic blood pressure (SBP), diastolic blood pressure (DBP) and pathological subtype (i.e., bacterial infection, non-bacterial infection and non-infection). Hypertension was defined as a blood pressure ≥140/90 mmHg or a history of antihypertensive treatment. This study was approved by the ethics committee of The First Hospital of Jilin University. All participants provided written informed consent.

### Measurements

**Fig 1:**
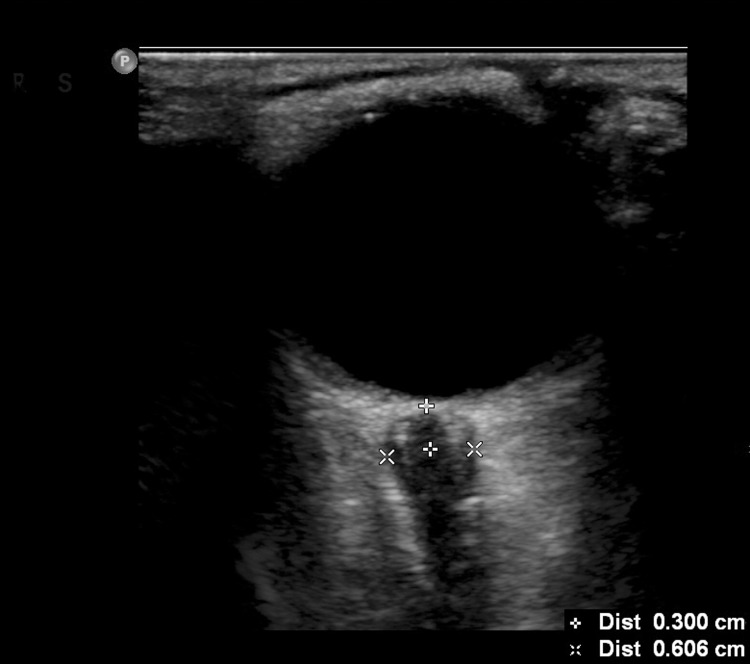
ONSD measurement. The ONSD measurement was assessed 3 mm posterior to the orbit. The ONSDs of the patient with increased ICPs were significantly enlarged.

The ONSD measurements were performed prior to the LPs. The interval between these two examinations was less than 10 min. The ultrasonographic procedure was performed by a senior emergency medicine resident. Ultrasound examinations of the eye were performed in B-mode using a Philips iU22 (Andover, Massachusetts, USA) ultrasound system and a 9–3 MHz linear array transducer. The patients were examined in the supine position. The probe was placed lightly on the closed upper eyelid with a thick layer of ultrasound gel to prevent pressure from being exerted on the eye. The position of the probe was adjusted to clearly display the entry of the optic nerve into the globe. According to previous protocols, two measurements were performed for each optic nerve. The first measurement was performed in the sagittal plane with the probe in a vertical orientation, and the second measurement was performed the transverse plane with the probe in a horizontal orientation [6,20]. The ONSD was assessed bilaterally 3 mm posterior to the orbit [21,22] (Fig. 1). The measurements were repeated once for each eye, and a total of eight values were obtained. The final ONSD measurement value for each patient was derived from the average of the eight values to minimize variability. The opening pressure of the CSF was recorded in cm of water pressure (cm H_2_O) by LP, which was performed by a neurological resident who was experienced and blind to the ultrasonographic results. Patients were placed in a left lateral position with their hips and knees flexed and their heads as close to their knees as comfortably possible. The area around the lower back was prepared using an aseptic technique. We asked the patients to relax. Once the subarachnoid space had been entered, the patient straightened his/her legs, the opening pressure on LP was then recorded, and fluid samples were obtained. Elevated opening pressure on LP was defined as a pressure > 20 cm H_2_O [17,19]. Subsequently, the characteristic data of the ONSD measurement values and the pressures of the CSF measured through the LP were entered into the database.

### Statistical analyses

Continuous variables are presented as the mean ± the standard deviation (SD), and categorical variables are reported as the frequency and percentage. Linear regression analyses, *t*-tests and ANOVA tests were performed independently to determine the relationships between the variables and the ONSDs. Univariate analyses were performed to examine the changes in intracranial pressure that corresponded to a given change in ONSD. Multiple logistic regression was performed to reveal the factors that affected this association. A receiver operating characteristic (ROC) curve was generated to determine the optimal cut-off point. All data were analyzed using the SPSS software package (SPSS version 18.0), and p<0.05 (two-tailed) was set as the level of statistical significance.

## Results

In total, 279 subjects (56.3% males) were included in the study, and a total of 2232 ONSDs were measured. The mean age of the patients was 41.3 ± 15.1 years. Eighteen patients had bacterial infections, and 138 patients had non-bacterial infections. The non-bacterial infections included viral infection (N = 132), a fungal infection (N = 1), cerebral cysticercosis (N = 3) and neurosyphilis (N = 2). The remaining 123 patients were diagnosed as being free of infection and were diagnosed with peripheral neuropathy (N = 21), hydrocephalus (N = 6), cerebrovascular disease (N = 43), cerebral tumor (N = 17), primary headache (N = 8) and epilepsy (N = 26), or the diagnoses were uncertain (N = 2). Elevated opening pressure on LP was observed in 101 subjects, and normal opening pressure on LP was diagnosed in 178 patients. A comparison of the characteristic data between the elevated opening pressure and the normal opening pressure groups is presented in Table 1. Statistically significant differences were found in age, ONSD, BMI and pathological subtype. The ONSD of the elevated opening pressure on LP group (4.58±0.46 mm) was significantly higher than that of the normal opening pressure on LP group (3.55±0.38 mm, p<0.001).

**Table 1:** Comparison of the characteristics of the increased ICP and normal ICP groups.

Characteristic	Increased ICP group (n = 101)	Normal group (n = 178)	*P*-value
Male (n,%)	61 (60.4%)	96 (53.9%)	0.296
Age (Y)	37.32±13.01	43.59±15.79	0.001
ONSD (mm)	4.58±0.46	3.55±0.38	<0.001
BMI (kg/m^2^)	23.89±4.11	22.81±3.49	0.020
Waistline(cm)	80.66±11.34	78.76±9.78	0.141
Head circumference (cm)	55.45±1.69	55.14±2.04	0.203
SBP (mmHg)	122.78±16.74	125.71±18.32	0.187
DBP (mmHg)	78.89±9.47	80.51±11.57	0.232
Pathological subtype			<0.001
Bacterial infection	15 (14.9%)	3 (1.7%)	
Non-bacterial infection	59 (58.4%)	79 (44.4%)	
Non-infection	27 (26.7%)	96 (53.9%)	

*ICP* intracranial pressure, *BMI* body mass index, *ONSD* optic nerve sheath diameter, *SBP* systolic blood pressure, *DBP* diastolic blood pressure.

The univariate analyses revealed that ONSD measurement (p<0.001), age (p = 0.001), BMI (p = 0.022) and pathological subtype (p<0.001) were significantly correlated with elevated opening pressure on LP. Linear regression analyses, *t*-tests and ANOVA tests revealed that gender (p = 0.005), age (p<0.001), BMI (p<0.001), waistline (p = 0.002), head circumference (p = 0.008) and pathological subtype (p<0.001) were significantly correlated with ONSD. The mean ONSD of the patients with bacterial infections, non-bacterial infections and without infections were 4.745 mm (SD 0.597), 4.013 mm (SD 0.656) and 3.698 mm (SD 0.500), respectively.

We included the significant variables in the multiple logistic regression. After adjusting for gender, age, BMI, waistline, head circumference and pathological subtype, ONSD was the only significant independent predictor of elevated opening pressure on LP (p<0.001).

**Fig 2:**
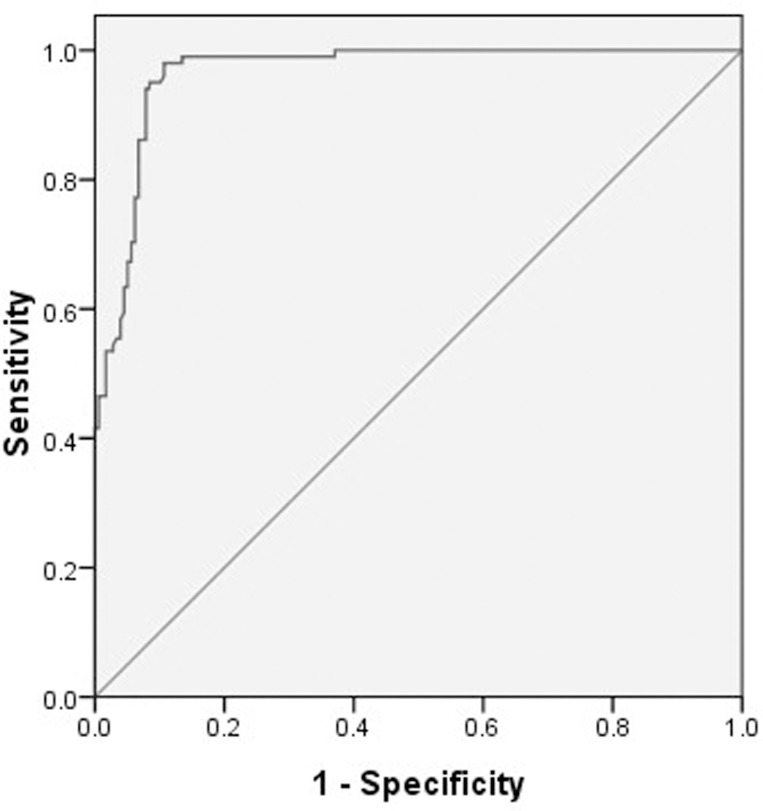
ROC curve for the optic nerve sheath diameter. AUC = 0.965 (95% CI: 0.947–0.984).

Using the opening pressure on LP as the standard criterion, we generated a ROC curve (Fig. 2) to determine the cut-off point that optimized the sensitivity and specificity. The ROC curve analysis revealed that the area under the curve (AUC) was 0.965 (95% CI: 0.947–0.984). The ONSD cut-off point for the identification of elevated opening pressure on LP was 4.1 mm, which yielded a sensitivity of 95% and a specificity of 92%.

## Discussion

The present research, which examined the ONSD and opening pressure on LP measurements of 279 patients, revealed that ultrasonographic ONSD might be a strong predictor of elevated opening pressure on LP with a high sensitivity and specificity compared with LP. We confirmed that ultrasonographic measurement of the ONSD was potentially useful for identifying patients with early elevated opening pressure on LP. Several previous studies have confirmed the reliability of the ONSD measurement in the evaluation of ICP, and we obtained similar results. Multivariate analysis revealed that the ONSD was an independent predictor for elevated opening pressure on LP. Although invasive ICP monitoring remains the gold standard for the establishment of ICP, it is not routinely used in many centers predominantly due to the lack of an available neurosurgeon to implant the monitoring device. Additionally, the total cost can be several thousand dollars. This study provides new information supporting the notion that ultrasonographic ONSD measurement is useful for rapid and safe screening for elevated opening pressure on LP when a patient is admitted into a hospital. This technique can provide an early indication of whether a patient requires invasive intracranial devices or transfer to a specialized center with an available neurosurgeon. Furthermore, ultrasonographic ONSD measurement is easy to learn and reproducible [23]. This technique is inexpensive and requires approximately 5 min per patient [17]. Moreover, this technique is reproducible with low intra- and inter-observer variation [24]. Thus, ultrasonographic ONSD measurement might be the preferred technique for patients who are suspected of increased ICP.

Despite the introduction of this technique two to three decades ago, increases in sonographic measures of the ONSD have not been properly evaluated in heterogeneous patients, particularly Asian patients. Currently, there are no definitive global standard values for the application of this procedure due to the small sample sizes (generally a few dozen) of the relevant studies. Compared with previous studies, our study had a relatively large sample size that included hundreds of patients.

The cut-off point for elevated opening pressure on LP found in our study is slightly different from the outcomes that have been obtained for Caucasian populations. In some western studies, the ONSD cut-off points for the diagnosis of increased ICP or elevated opening pressure on LP have ranged from 5.0 to 5.9 mm [7,17,25,26]. Recent studies found that ONSD detection of increased ICP was slightly lower than 5.0mm [9,27]. However, the cut-off point identified in the present study was markedly lower than 5.0 mm. There are several possible reasons for this result. First, ethnic differences or genetic distinctions might affect the cut-off point. The majority of the previous studies were performed in Caucasian populations, while our study targeted a Chinese population. Second, the subjects included in the majority of the previous studies had severe injuries or required treatment in the intensive care unit. In contrast, the subjects of our study were free of emergency statuses and were cared for in a general ward, which might have resulted in less severe increases in ICP being predicted based on the ONSD. Intensive care procedures, such as sedation or mechanical ventilation, have been inferred to be associated with ONSD alterations [28]. Furthermore, the degree of the dilation of the ONSD is greater due to dramatically increased ICP. Some animal models have shown that the ONSD increases at a rate of approximately 0.0034 mm per 1 mm Hg increase in ICP [29]. In isolated human optic nerves obtained from autopsies, an additional ONS dilatation of 0.25 mmHg resulted from every 10 mmHg pressure increase [30]. Thus, the discrepancy regarding the cut-off point identified in our study might be attributable to these aforementioned reasons.

Because we aimed to identify patients in the early stages of increased ICP, the findings of our study were closer to the critical point of increased ICP. Due to the features of the patients who were included in our study, we obtained the measurements at very early stages of increased ICP. Because elevated ICP is associated with poor outcomes, timely detection in the early stage plays an important role in clinical practice.

Several studies of normal values have reported a relatively broad interindividual range of ONSD measurements [31], [28]. A protocol used by an ONSD research group in 2013 proposed to determine whether the diagnostic accuracy of ONSD ultrasonography varies according to patient characteristics (e.g., age, weight, etc.)[18]. Nevertheless, to the best of our knowledge, there have been no publications that have confirmed whether some interindividual factors affect the use of ultrasonographic ONSD measurements as a screening tool for increased ICP. To some extent, our study addresses this research gap. Gender, age, BMI, waistline, head circumference, blood pressure and pathological subtype were found to lack significant influences on the relationship between ONSD and opening pressure on LP in our study. In the last few years, neuroimaging of the ONSD with computed tomography (CT) scans and MRI have been found to be potentially useful in the detection of increased ICP [32,33]. A recent study about OSND in Caucasians using CT scans found the measurement of OSND at the 3mm point was more variable and the most stable measurement values could be obtained if the ONSD was measured at 10 mm from globe. The authors of that study suggested that the optic nerve/eyeball diameter index was much less variable than the ONSD and might be used for ICP monitoring [34]. Hence, further studies should focus on comparisons of ultrasonography with CT scan and MRI ONSD measurements. Additionally, the influence of different measurement depths and other factors that could potentially influence this association should be investigated.

Our study has some limitations. First, although the final ONSD measurement value for each patient in our study was derived from an average of eight values to minimize variability, technical factors, such as angle of the probe, the patient’s position and the measured areas of the ONSD, might have affected the measured values. Second, LP is not truly equivalent to the gold standard of intracranial pressure monitoring. The opening pressure on LP might be altered by patient anxiety, position, muscle tone and pressure on the abdomen. Third, a more accurate cut-off point is required to enable the use of ultrasonographic ONSD measurement as a widely available and reliable technique for the identification of patients who are at risk for increased ICP. Multi-center studies with larger sample sizes should be undertaken in the future.

## Conclusion

The ONSD measurement cut-off point for the prediction of elevated opening pressure on LP in Chinese patients was less than that of Caucasian patients. Thus, we propose that ethnic differences should be noted and appropriately applied to the corresponding ultrasonographic criteria.

## References

[1] Juul N, Morris GF, Marshall SB, Marshall LF (2000) Intracranial hypertension and cerebral perfusion pressure: influence on neurological deterioration and outcome in severe head injury. Journal of neurosurgery 92: 1–6. 10616075 10.3171/jns.2000.92.1.0001

[2] Balestreri M, Czosnyka M, Hutchinson P, Steiner LA, Hiler M, et al (2006) Impact of intracranial pressure and cerebral perfusion pressure on severe disability and mortality after head injury. Neurocritical care 4: 8–13. 16498188 10.1385/NCC:4:1:008

[3] Lundberg N (1959) Continuous recording and control of ventricular fluid pressure in neurosurgical practice. Acta psychiatrica Scandinavica Supplementum 36: 1–193. 13764297

[4] Raboel P, Bartek J, Andresen M, Bellander B, Romner B (2012) Intracranial pressure monitoring: invasive versus non-invasive methods—a review. Critical care research and practice 2012 10.1155/2012/964547 22720148 PMC3376474

[5] Kristiansson H, Nissborg E, Bartek J Jr, Andresen M, Reinstrup P, et al (2013) Measuring elevated intracranial pressure through noninvasive methods: a review of the literature. Journal of neurosurgical anesthesiology 25: 372–385. 10.1097/ANA.0b013e31829795ce 23715045

[6] Geeraerts T, Launey Y, Martin L, Pottecher J, Vigué B, et al (2007) Ultrasonography of the optic nerve sheath may be useful for detecting raised intracranial pressure after severe brain injury. Intensive care medicine 33: 1704–1711. 17668184 10.1007/s00134-007-0797-6

[7] Soldatos T, Chatzimichail K, Papathanasiou M, Gouliamos A (2009) Optic nerve sonography: a new window for the non-invasive evaluation of intracranial pressure in brain injury. Emergency Medicine Journal 26: 630–634. 10.1136/emj.2008.058453 19700575

[8] Moretti R, Pizzi B (2011) Ultrasonography of the optic nerve in neurocritically ill patients. Acta Anaesthesiologica Scandinavica 55: 644–652. 10.1111/j.1399-6576.2011.02432.x 21463263

[9] Rajajee V, Vanaman M, Fletcher JJ, Jacobs TL (2011) Optic nerve ultrasound for the detection of raised intracranial pressure. Neurocritical care 15: 506–515. 10.1007/s12028-011-9606-8 21769456

[10] Hansen H, Helmke K (1996) The subarachnoid space surrounding the optic nerves. An ultrasound study of the optic nerve sheath. Surgical and Radiologic Anatomy 18: 323–328. 8983112 10.1007/BF01627611

[11] Hayreh SS (1968) Pathogenesis of oedema of the optic disc. Documenta Ophthalmologica 24: 289–411. 4972870 10.1007/BF02550944

[12] Liu D, Kahn M (1993) Measurement and relationship of subarachnoid pressure of the optic nerve to intracranial pressures in fresh cadavers. American journal of ophthalmology 116: 548–556. 8238213 10.1016/s0002-9394(14)73195-2

[13] Hansen H-C, Helmke K (1997) Validation of the optic nerve sheath response to changing cerebrospinal fluid pressure: ultrasound findings during intrathecal infusion tests. Journal of neurosurgery 87: 34–40. 9202262 10.3171/jns.1997.87.1.0034

[14] Newman W, Hollman A, Dutton G, Carachi R (2002) Measurement of optic nerve sheath diameter by ultrasound: a means of detecting acute raised intracranial pressure in hydrocephalus. British journal of ophthalmology 86: 1109–1113. 12234888 10.1136/bjo.86.10.1109 PMC1771326

[15] Blaivas M, Theodoro D, Sierzenski PR (2003) Elevated intracranial pressure detected by bedside emergency ultrasonography of the optic nerve sheath. Academic emergency medicine 10: 376–381. 12670853 10.1111/j.1553-2712.2003.tb01352.x

[16] Tsung JW, Blaivas M, Cooper A, Levick NR (2005) A rapid noninvasive method of detecting elevated intracranial pressure using bedside ocular ultrasound: application to 3 cases of head trauma in the pediatric emergency department. Pediatric emergency care 21: 94–98. 15699817 10.1097/01.pec.0000159052.64930.64

[17] Kimberly HH, Shah S, Marill K, Noble V (2008) Correlation of optic nerve sheath diameter with direct measurement of intracranial pressure. Academic Emergency Medicine 15: 201–204. 10.1111/j.1553-2712.2007.00031.x 18275454

[18] Dubourg J, Messerer M, Karakitsos D, Rajajee V, Antonsen E, et al (2013) Individual patient data systematic review and meta-analysis of optic nerve sheath diameter ultrasonography for detecting raised intracranial pressure: protocol of the ONSD research group. Systematic reviews 2: 1–6. 10.1186/2046-4053-2-1 23919384 PMC3751128

[19] Adams RD, Victor M (2001) Principles of Neurology (7th ed). McGraw-Hill New York, NY.

[20] Moretti R, Pizzi B, Cassini F, Vivaldi N (2009) Reliability of optic nerve ultrasound for the evaluation of patients with spontaneous intracranial hemorrhage. Neurocritical care 11: 406–410. 10.1007/s12028-009-9250-8 19636971

[21] Tayal VS, Neulander M, Norton HJ, Foster T, Saunders T, et al (2007) Emergency department sonographic measurement of optic nerve sheath diameter to detect findings of increased intracranial pressure in adult head injury patients. Annals of emergency medicine 49: 508–514. 16997419 10.1016/j.annemergmed.2006.06.040

[22] Romagnuolo L, Tayal V, Tomaszewski C, Saunders T, Norton HJ (2005) Optic nerve sheath diameter does not change with patient position. The American journal of emergency medicine 23: 686–688. 16140179 10.1016/j.ajem.2004.11.004

[23] Potgieter D, Kippin A, Ngu F, McKean C (2011) Can accurate ultrasonographic measurement of the optic nerve sheath diameter (a non-invasive measure of intracranial pressure) be taught to novice operators in a single training session? Anaesthesia and intensive care 39: 95–100. 21375098 10.1177/0310057X1103900116

[24] Ballantyne S, O'Neill G, Hamilton R, Hollman A (2002) Observer variation in the sonographic measurement of optic nerve sheath diameter in normal adults. European journal of ultrasound 15: 145–149. 12423741 10.1016/s0929-8266(02)00036-8

[25] Dubourg J, Javouhey E, Geeraerts T, Messerer M, Kassai B (2011) Ultrasonography of optic nerve sheath diameter for detection of raised intracranial pressure: a systematic review and meta-analysis. Intensive care medicine 37: 1059–1068. 10.1007/s00134-011-2224-2 21505900

[26] Amini A, Kariman H, Arhami Dolatabadi A, Hatamabadi HR, Derakhshanfar H, et al (2013) Use of the sonographic diameter of optic nerve sheath to estimate intracranial pressure. The American journal of emergency medicine 31: 236–239. 10.1016/j.ajem.2012.06.025 22944553

[27] Maude RR, Hossain MA, Hassan MU, Osbourne S, Sayeed KLA, et al (2013) Transorbital Sonographic Evaluation of Normal Optic Nerve Sheath Diameter in Healthy Volunteers in Bangladesh. PloS one 8: e81013 10.1371/journal.pone.0081013 24312515 PMC3846670

[28] Bäuerle J, Lochner P, Kaps M, Nedelmann M (2012) Intra‐and Interobsever Reliability of Sonographic Assessment of the Optic Nerve Sheath Diameter in Healthy Adults. Journal of Neuroimaging 22: 42–45. 10.1111/j.1552-6569.2010.00546.x 21121998

[29] Hamilton DR, Sargsyan AE, Melton SL, Garcia KM, Oddo B, et al (2011) Sonography for determining the optic nerve sheath diameter with increasing intracranial pressure in a porcine model. Journal of Ultrasound in Medicine 30: 651–659. 21527613 10.7863/jum.2011.30.5.651

[30] Hansen HC, Lagreze W, Krueger O, Helmke K (2011) Dependence of the optic nerve sheath diameter on acutely applied subarachnoidal pressure–an experimental ultrasound study. Acta ophthalmologica 89: e528–e532. 10.1111/j.1755-3768.2011.02159.x 21518306

[31] Lagrèze WA, Lazzaro A, Weigel M, Hansen H-C, Hennig J, et al (2007) Morphometry of the retrobulbar human optic nerve: comparison between conventional sonography and ultrafast magnetic resonance sequences. Investigative ophthalmology & visual science 48: 1913–1917. 10.1016/j.jaad.2014.08.024 17460241

[32] Geeraerts T, Newcombe VF, Coles JP, Abate MG, Perkes IE, et al (2008) Use of T2-weighted magnetic resonance imaging of the optic nerve sheath to detect raised intracranial pressure. Crit Care 12: R114 10.1186/cc7006 18786243 PMC2592740

[33] Legrand A, Jeanjean P, Delanghe F, Peltier J, Lecat B, et al (2013) Estimation of optic nerve sheath diameter on an initial brain computed tomography scan can contribute prognostic information in traumatic brain injury patients. Crit Care 17: R61 10.1186/cc12589 23536993 PMC3672708

[34] Vaiman M, Gottlieb P, Bekerman I (2014) Quantitative relations between the eyeball, the optic nerve, and the optic canal important for intracranial pressure monitoring. Head Face Med 10: 32 10.1186/1746-160X-10-32 25130267 PMC4141911

